# The Effects of Paleoclimatic Events on Mediterranean Trout: Preliminary Evidences from Ancient DNA

**DOI:** 10.1371/journal.pone.0157975

**Published:** 2016-06-22

**Authors:** Andrea Splendiani, Tatiana Fioravanti, Massimo Giovannotti, Alessandra Negri, Paolo Ruggeri, Luigi Olivieri, Paola Nisi Cerioni, Massimo Lorenzoni, Vincenzo Caputo Barucchi

**Affiliations:** 1 Dipartimento di Scienze della Vita e dell'Ambiente (DiSVA), Università Politecnica delle Marche, Via Brecce Bianche, 60131 Ancona, Italy; 2 Dipartimento di Chimica, Biologia e Biotecnologie, Università di Perugia, Via Elce di Sotto, 06123 Perugia, Italy; 3 Istituto Italiano di Paleontologia Umana, Is.I.P.U., Convitto Nazionale Regina Margherita, Piazza Ruggero Bonghi 2. 03012 Anagni, Italy; Swansea University, UNITED KINGDOM

## Abstract

In this pilot study for the first time, ancient DNA has been extracted from bone remains of *Salmo trutta*. These samples were from a stratigraphic succession located in a coastal cave of Calabria (southern Italy) inhabited by humans from upper Palaeolithic to historical times. Seven pairs of primers were used to PCR-amplify and sequence from 128 to 410 bp of the mtDNA control region of eleven samples. Three haplotypes were observed: two (ADcs-1 and MEcs-1) already described in rivers from the Italian peninsula; one (ATcs-33) belonging to the southern Atlantic clade of the AT *Salmo trutta* mtDNA lineage (*sensu* Bernatchez). The prehistoric occurrence of this latter haplotype in the water courses of the Italian peninsula has been detected for the first time in this study. Finally, we observed a correspondence between frequency of trout remains and variation in haplotype diversity that we related with ecological and demographic changes resulting from a period of rapid cooling known as the Younger Dryas.

## Introduction

The Pleistocene (from 2.58 to 0.0117Ma, [1]) is a geological epoch characterized by repeated glacial-interglacial cycles that caused drastic environmental changes and impacted the distribution range and the genetic diversity of species and populations [2]. During glacial periods, the expansion of ice sheets forced north-temperate species to move southward and survive in warmer refugia, the same species moved northwards in interglacial phases to recolonize their previous habitat. This process lead to “southern richness and northern purity” in north-temperate species biodiversity [2, 3]. Species unable to shift their distribution range or adapt to the new environmental conditions became extinct at local or global scale [4, 5].

Despite freshwater ecosystems cover only 0.8% of the Earth’s surface, they host slightly less than one-half of the fish species currently known [6]. This abundance of species in a so limited and confined space makes freshwater biodiversity particularly sensitive to various threats such as: i) climate changes; ii) overexploitation; iii) pollution; iv) flow modification; v) habitat degradation; and vi) invasion of exotic species [7]. One major threat is represented by the rapid and continuous global warming phase our planet is going through [8] that could lead to an inexorable decline of biodiversity over the 21st century. Probably, freshwater species will be the most affected [9, 10]. Indeed, according to Xenopoulos et al. [11], over 75% of fish biodiversity will be lost in the next 70 years in riverine systems due to reduced discharge. Besides the alteration of river’s flow, climate change will threaten the survival of freshwater fish species because of the increase in water temperature and the subsequent reduction of dissolved oxygen [12]. Among freshwater fishes, salmonids are probably the organisms most sensitive to the severe alterations in the environmental parameters mentioned above [13]. Therefore, to understand how salmonids reacted to past climate changes is important to predict how these fishes will respond to the future impact of climate changes on freshwater ecosystems.

Climatic fluctuations and environmental changes that occurred during the Pleistocene seem to have influenced the present distribution range of many salmonids [14]. Therefore, the *Salmo trutta* species complex could be considered a good model to study how paleoclimatic changes affected biological and ecological traits of these freshwater fishes. This species complex is distributed largely across Eurasia and North Africa with anadromous populations occurring throughout its distribution range, except in the Mediterranean basin [15, 16]. Several mitochondrial DNA (mtDNA) studies showed that Pleistocene successions of glacial and interglacial periods determined the conditions for the evolution of six *S*. *trutta* lineages: Atlantic (AT), Danubian (DA), Mediterranean (ME), Adriatic (AD), *marmoratus* (MA) and Duero (DU) lineages [17– 19].

Genetic characterization of Pleistocene salmonids could provide crucial information concerning phylogeographic issues and temporal change in demographic and life history characteristics [20, 21]. Over the last 30 years, great developments in the study of DNA from historical and archaeological samples have been made [22]. This led to use ancient DNA (aDNA) as a useful tool to perform retrospective monitoring of genetic biodiversity [23]. The major studies in this field of research are focused on response of mammal populations during Pleistocene-Holocene climatic change [24–26], especially to assess the causes of Quaternary megafauna extinctions [27]. Much less has been done to reveal past climate change effects on fish species [20, 21]. Indeed, aDNA studies on fish species were centered mainly on taxonomic identification of archaeological remains, without considering the role of climate changes in fish micro-evolutionary pathways [28–32].

Fishing and consumption of freshwater species are known for Europe during prehistoric times, when opportunistic Upper-Paleolithic hunter-gatherers fed mainly on large mammals, but integrated their diet also with birds, mollusks and fishes [33]. Among these alternative food sources freshwater fishes were the most common, especially salmonids, as demonstrated by archaeological excavations [34–37]. Therefore, trout and/or salmon bone remains accumulated in archaeological sites represent valuable records to study how past climate oscillations affected fish populations (e.g., [38]). In the sedimentary succession in the *Grotta del Santuario della Madonna* (Praia a Mare, Cosenza, Italy, GSM henceforth), described by Cardini [39], *Salmo trutta* remains were found along a stratigraphic succession spanning from the Upper Paleolithic to the Mesolithic [40]. The distinctive vomer bones allowed the identification of these remains as belonging to *S*. *trutta*. In addition, the coastal position of the cave and the bone size led to hypothesize that these *S*. *trutta* remains belonged to anadromous individuals [40]. According to the literature [41], the sedimentary succession in GSM displays a continuous series of remains referable to the presence of a human settlement from the late Pleistocene to the Holocene. In this succession, three different climatic phases can be recognised: i) the Bølling-Allerød interstadial, a warm period at the end of the Pleistocene (*c*. 14,700–12,700 cal yr BP), ii) the Younger Dryas (YD) stadial, a period that lasted 1,300 (±70) yr, characterized by cold climatic conditions and droughts, which occurred between approximately 12,800 and 11,600 cal yr BP, and iii) the Preboreal oscillation of the early Holocene (*c*. 11,500 cal yr BP).

In this study, bone remains of trout were selected from the stratigraphic succession of GSM. aDNA was extracted from these remains and the variation of a portion of the mtDNA control region (D-loop) was analysed in order to verify if genetic diversity changes occurred in the period of time encompassed by the stratigraphic succession analysed. The mtDNA genetic diversity observed was then compared with *S*. *trutta* abundance [40] and paleoclimatic reconstructions.

## Materials and Methods

**Fig 1:**
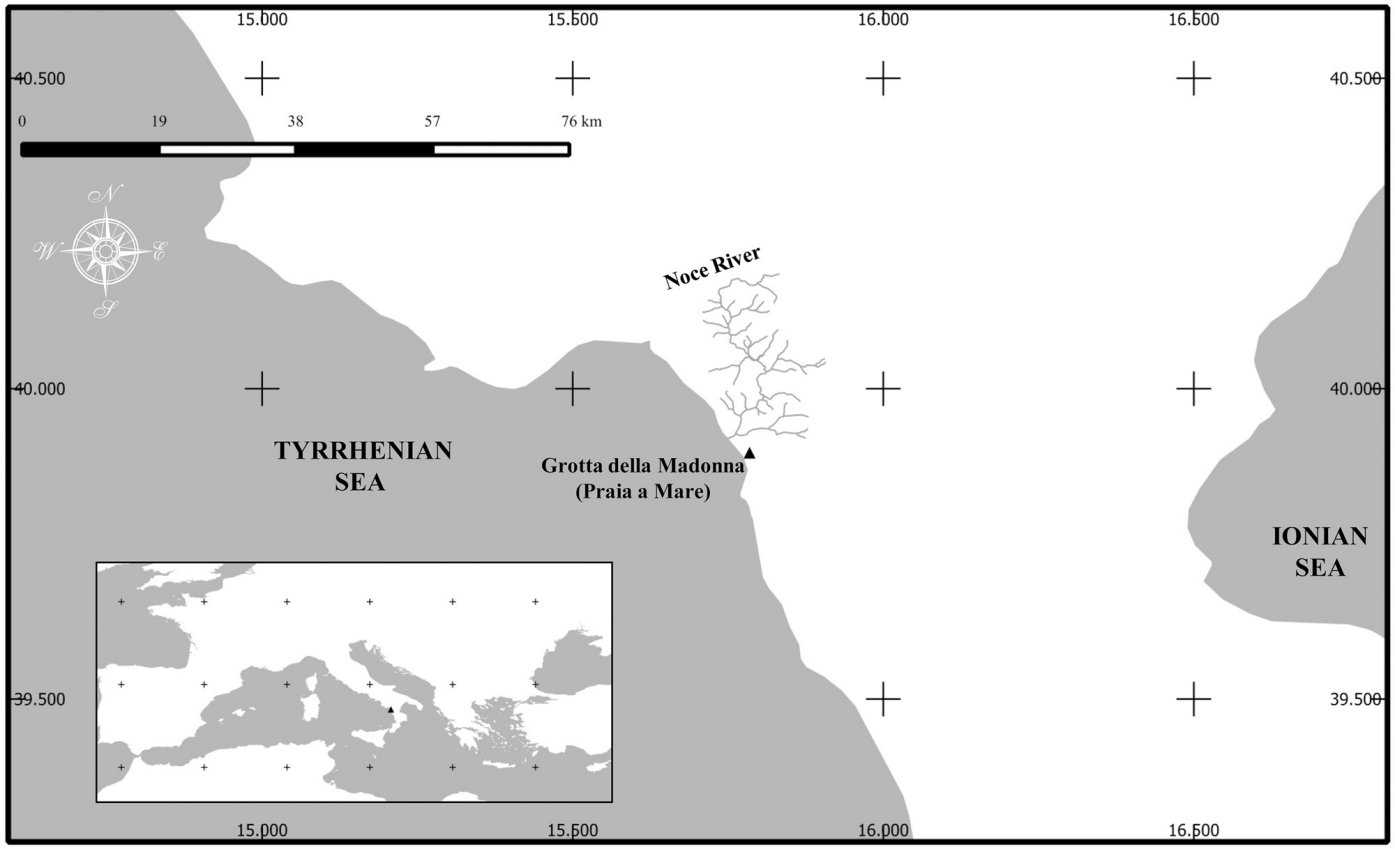
Geographic position of Grotta del Santuario della Madonna.

*Salmo trutta* bone samples used in this study were collected between 1957 and 1970 during the excavation of GSM [39] and provided to us by the *Istituto Italiano di Paleontologia Umana (Is*.*I*.*P*.*U*.*)*. A total of 12 brown trout sub-fossil bone remains were collected for this study. This brown trout bone collection is housed at the storage facilities of *Is*.*I*.*P*.*U*., [Piazza Ruggero Bonghi 2, 03012 Anagni (FR), Italy]. The permit for studying and using the above samples was issued by Fabio Parenti of the Scientific Comittee of Istituto Italiano di Paleontologia Umana, on March 14th, 2014. This cave is located on the slopes of Monte Vingiolo, Praia a Mare village (Cosenza, Italy), at *c*. 50 m above sea level and not far from the mouth of the Noce River (Fig 1). The excavation exposed a stratigraphic succession about 12 m thick that contained several faunal and artifact remains. The finding shows that GSM was occupied by humans at least from the late Upper Paleolithic (around 14,200 cal yr BP) to historical times. This stratigraphic succession was divided into ten distinct archaeological layers which correspond to different periods when the cave was inhabited, as reported by Alessio et al., [42, 43], from top to bottom: I, Roman period; II, Bronze age; III, Eneolithic; IV, period not identified; V, late Neolithic; VI, VII and VIII, Middle Neolithic; IX, Mesolithic and X, Upper Paleolithic. Each layer was subdivided into stratigraphical spits (or sections) numbered with Arabic numbers from 0 (top) to 72 (bottom) and, for some of them, samples of charcoal, charred bones or shells were radiocarbon dated by Alessio et al. [42, 43]. To establish the correct temporal occurrence of our samples, radiocarbon dates estimated for sections from which the samples were obtained were calibrated using the CalPal software [44], CalCurve: CalPal2007HULU (for charcoal and charred bones) and INTCAL 04 Marine [45] (for marine shells). This work was focused only on the Upper Paleolithic layer (X) and a part of the Mesolithic one (IX) in which a lot of *S*. *trutta* remains (8,290 specimens) were found, especially concentrated in the sections from 65 to 39 [40].

**Fig 2:**
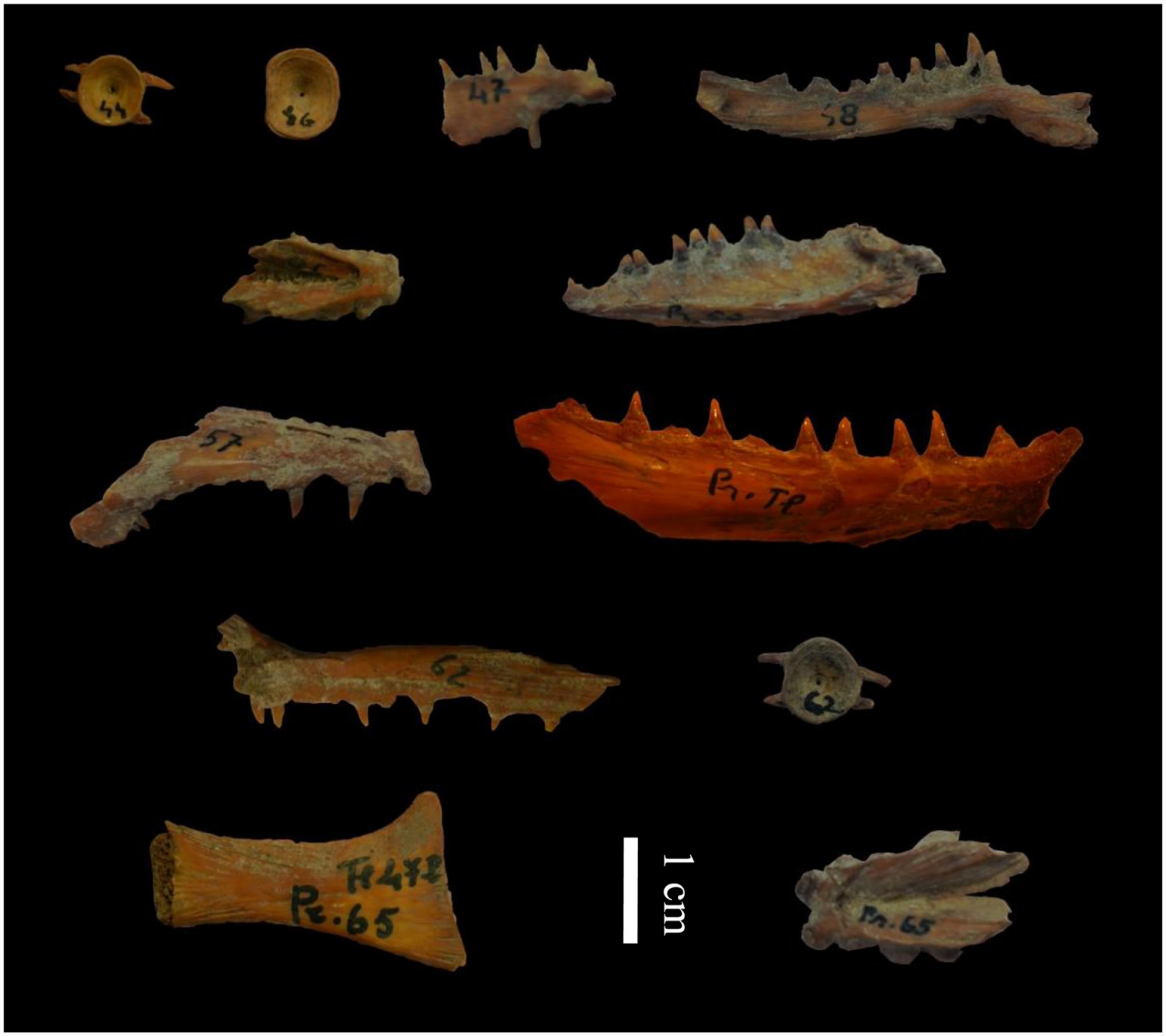
*Salmo trutta* sub-fossil samples analysed in this study.

**Fig 3:**
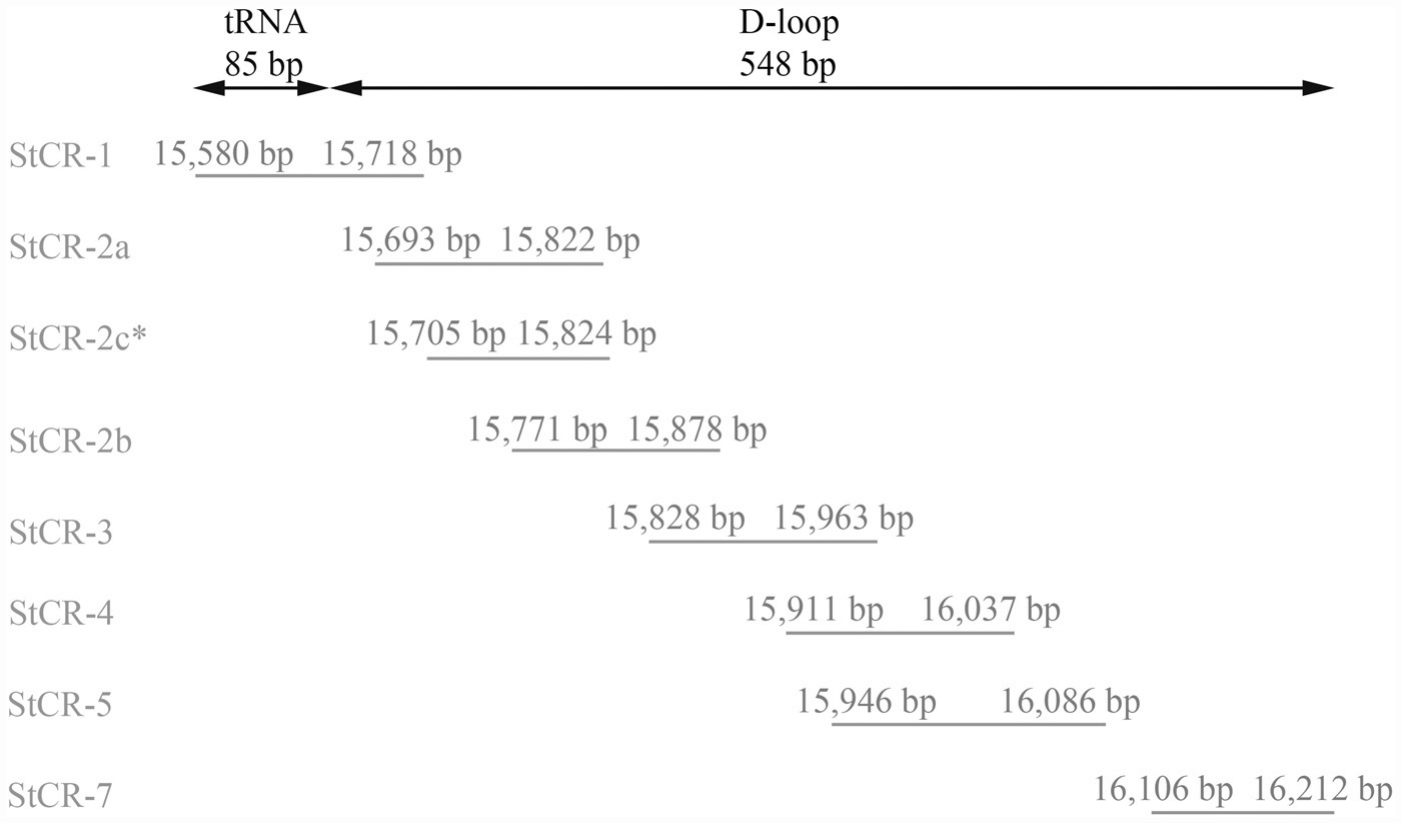
Primer pairs designed and used in this study. Primer pairs were designed on the basis of the brown trout complete mitochondrial genome present in GenBank (Accession no. AM910409.1) to amplify a 633 bp fragment of the mtDNA, including 85 bp of tRNA Pro-gene and 548 bp of mtDNA D-loop. *StCR-2c primer pair was designed to substitute for StCR-2a in the cases in which the amplification with the latter pair of primers was not successful.

A total of 12 *Salmo trutta* bone samples (Fig 2 and Table 1) were selected from eight stratigraphical sections. During laboratory analyses the bone remains were divided into two groups (six bones per group) and then, for each group, DNA extraction, PCR amplification and sequencing were performed in two distinct periods of time. This was done to test the validity of results. Either half of a large bone or a whole small bone was processed for aDNA extraction using a modified silica-spin column method [29]. The initial physical-chemical decontamination step was modified as proposed by Speller et al. [30], and the bone powder from each sample was subdivided to have three extraction replicas. After the DNA extraction, a 633 bp fragment of the mitochondrial DNA, including 85 bp of tRNA Pro-gene and 548 bp of mtDNA D-loop, was amplified using six overlapping pairs of primers (Fig 3 and Table 2). An additional primer set (StCR-7) was designed to amplify a consecutive but not overlapped D-loop region with a mutation characteristic of the South European Atlantic clade of *S*. *trutta* (i.e. AT3-3 clade *sensu* Cortey et al. [19]).

**Table 1:** Information for *Salmo trutta* bone samples analysed.

Layer	Section	Sample ID	Laboratory ID	Sample type
**First set of samples analysed**				
IX	44	44	V44	Vertebra
IX	46	46	V46	Vertebra
X	50	50	M50	Glossohyal
X	57	Pr-Tl57	PT57	Dentary
X	62	62	M62	Maxilla
X	65	Tl472 Pr-65	TP65	Ceratohyal
**Second set of samples analysed**				
IX	47	47	M47	Maxilla
X	48	48	M48	Maxilla
X	50	Pr50	Pr50	Dentary
X	57	57	M57	Maxilla
X	62	62	V62	Vertebra
X	65	Pr65	Pr65	Glossohyal

**Table 2:** Details regarding primer pairs used in this study.

Primer	Sequence 5' to 3'	Product length (bp)
**StCR-1**		
**F**	AACCCTCCCTAGTGCTCAGA **(20)**	139
**R**	TATAACATTGGGTTAGCAAGGTACA **(25)**	
**StCR-2a**		
**F**	TTGTACCTTGCTAACCCAATGTT **(23)**	130
**R**	GGGGTTAAATTCACTAATGTTGA **(23)**	
**StCR-2b**		
**F**	AGCATGTGAGTAGTACATCAT **(21)**	108
**R**	TGGTTATTATCACGTGTTTTGCT **(23)**	
**StCR-2c**		
**F**	AACCCAATGTTATACTACATCTAT **(24)**	120
**R**	GAGGGGTTAAATTCACTAATGTTGA **(25)**	
**StCR-3**		
**F**	CATCAGCACTAACTCAAGGTTTACA **(25)**	136
**R**	TGCTGAAAGTTGGTGGGTAA **(20)**	
**StCR-4**		
**F**	TCAATAAAACTCCAGCTACACG **(23)**	127
**R**	TGTCCCTGACCATCAATAAGAG **(22)**	
**StCR-5**		
**F**	ACCCACCAACTTTCAGCATC **(20)**	141
**R**	GGAACCAAATGCCAGGAATA **(20)**	
**StCR-7**		
**F**	AAGAAACCACCCCCTGAAAG **(20)**	107
**R**	GGGAACCCTATGCATATAA **(19)**	

Each mtDNA fragment was amplified for every sample in a separate 25 μl PCR reaction containing 1X PCR buffer, 1.5 mM MgCl_2_, 0.08 mM of each dNTP, 0.48 μM of each primer, 4 U Platinum Taq DNA Polymerase (Invitrogen) and 3 μl of DNA extract. PCR was performed in a BIO RAD T100 Thermal Cycler with a 7 min initial denaturation at 94°C, followed by 60 cycles at 94°C for 20 s (denaturation), 53.9°C for 30 s (annealing), 72°C for 40 s (extension), and a final extension at 72°C for 7 min. All amplicons were visualized on 2% agarose gels to evaluate success and quality of amplification. PCR products were purified by EXOSAP and Sanger sequenced with both forward and reverse primers using an ABIPRISM 3730XL DNA sequencer. To validate our results, PCR amplification and sequencing were performed twice, from different extraction replicas of each sample. To avoid contamination, DNA extraction and PCR preparation were performed in a dedicated laboratory [46, 47], in which modern trout DNA had never been introduced. Furthermore, the setting of the laboratory and the preparation of all working phases followed many of the recommendations from Knapp et al. [48] to prevent DNA contamination. Each step was performed in two different laboratory hoods in order to spatially separate extraction and PCR preparation phases. Each sample was manipulated and processed individually from the DNA extraction to sequencing with no overlapping steps in samples handling. Other precautions to prevent human-DNA contamination were adopted like wearing full body disposable suits, laboratory dedicated shoes, face masks and gloves. All working surfaces were washed with 10% bleach and UV-lighted for 20–30 min before and after any work session. Dedicated pipettes, filtered pipette tips, laboratory plasticware, glassware and reagents, were irradiated with UV in the same way as the working surfaces. In order to avoid the use of thermal cycler already used for PCR amplifications of modern trout DNA, a separate room for PCR was provided with a brand new thermal cycler that was cleaned before every amplification with 10% bleach and, after some minutes, rinsed with pure sterile water. To detect any possible contamination, extraction and PCR-negative controls were performed in all cases [46].

The sequences of all fragments were assembled to give a partial mtDNA control region sequence. The assembled sequences were first analysed by BLAST [49] to obtain preliminary taxonomic identifications. Subsequently, aDNA sequences obtained in this study were aligned with six modern *Salmo trutta* sequences found in GenBank using CLUSTALW [50, 51]. The classification into the main brown trout mtDNA lineages was based i) on the diagnostic nucleotide state of character observed in polymorphic sites and ii) a haplotype network based on the 410 bp mitochondrial DNA data set composed of 125 modern (see S1 Table) and our three ancient haplotypes using the TCS method [52] in PopART v1.7 [53].

## Results

### Genetic analyses

After PCR amplification and sequencing of seven fragments a total of 410 bp at the 5' end of the mtDNA control region was successfully reconstructed for six samples out of 12. For five samples, we were able to amplify from three to six of the seven fragments, while for one sample we failed in all PCR attempts (S2 Table). The samples with the highest number of missing amplified fragments were the most recent one (V44) and the two oldest (TP65 and Pr65) (see Table 3 and S2 Table). The worst amplification success was observed for fragment 2a. In this case the use of a new primer set (StCR-2c) allowed the amplification of a region shorter than 2a (Fig 3 and S2 Table).

**Table 3:** Alignment result.

	15,690	15,775	15,809	15,859	15,925	16,052	16,053	16,066	16,193
**ATcs-1(AF273086)**	**T**	**T**	**G**	**A**	**G**	**C**	**T**	**T**	**C**
**ATcs-3(AF274574)**	.	.	.	.	.	.	.	.	**T**
**ATcs-25(EF530487)**	.	.	**A**	.	.	.	.	.	**T**
**ATcs-33(EF530495)**	.	**C**	**A**	.	.	.	.	.	.
V44(KX022957)	-	-	-	-	-	.	.	.	-
V46(KX022958)	.	C	A	.	.	.	.	.	.
M47(KX022959)	-	C	A	.	.	.	.	.	.
M48(KX022960)	.	C	A	.	.	.	.	.	.
Pr50(KX022961)	.	C	A	.	.	.	.	.	.
M50(KX022962)	.	C	A	.	.	.	.	.	.
**ADcs-1(AY836330)**	**C**	.	.	.	**C**	**T**	**C**	**C**	**T**
PT57(KX022963)	C	-	.	.	C	T	C	C	T
**MEcs-1(AY836350)**	**C**	.	**A**	**C**	.	**T**	**C**	**C**	**T**
M57(KX022964)	C	.	A	C	.	T	C	C	T
M62(KX022965)	C	.	A	C	.	T	C	C	T
V62(KX022966)	C	.	A	C	.	T	C	C	T
TP65(KX022967)	.	C	A	-	-	.	.	.	.
Pr65	-	-	-	-	-	-	-	-	-

Diagnostic sites are numbered with reference to the brown trout complete mitochondrial genome (GenBank accession no. AM910409.1) and were obtained by aligning our ancient D-loop sequences against reference sequences from GenBank (in bold). GenBank accession numbers are in brackets. Dashes (-) indicate missing data due to sequencing failure.

The BLAST search performed for all the sequences obtained showed that all the bone remains analysed here belong to *S*. *trutta* individuals, consistent with morphological identification of bone samples carried out by Durante [40]. The comparison with modern brown trout D-loop sequences found in GenBank highlighted the presence of nine variable positions, which allowed the identification of three different haplotypes already described in the literature: MEcs1, ADcs1 [54] and ATcs-33 [19] (Table 3). The oldest sample TP65 was identified as the ATcs-33 haplotype on the basis of the state of character observed at the nucleotide positions 15,775, 15,809 and 16,193 of the sequence assembled from the five segments amplified. The classification of the sample V44 was more difficult due to the presence of only three variable sites (positions 16,052, 16,053 and 16,066) which allowed its attribution to the Atlantic lineage but not to a specific haplotype. However, due to the observation of the sole haplotype ATcs-33 in the rest of the Atlantic samples we also attributed this haplotype to the sample V44.

**Fig 4:**
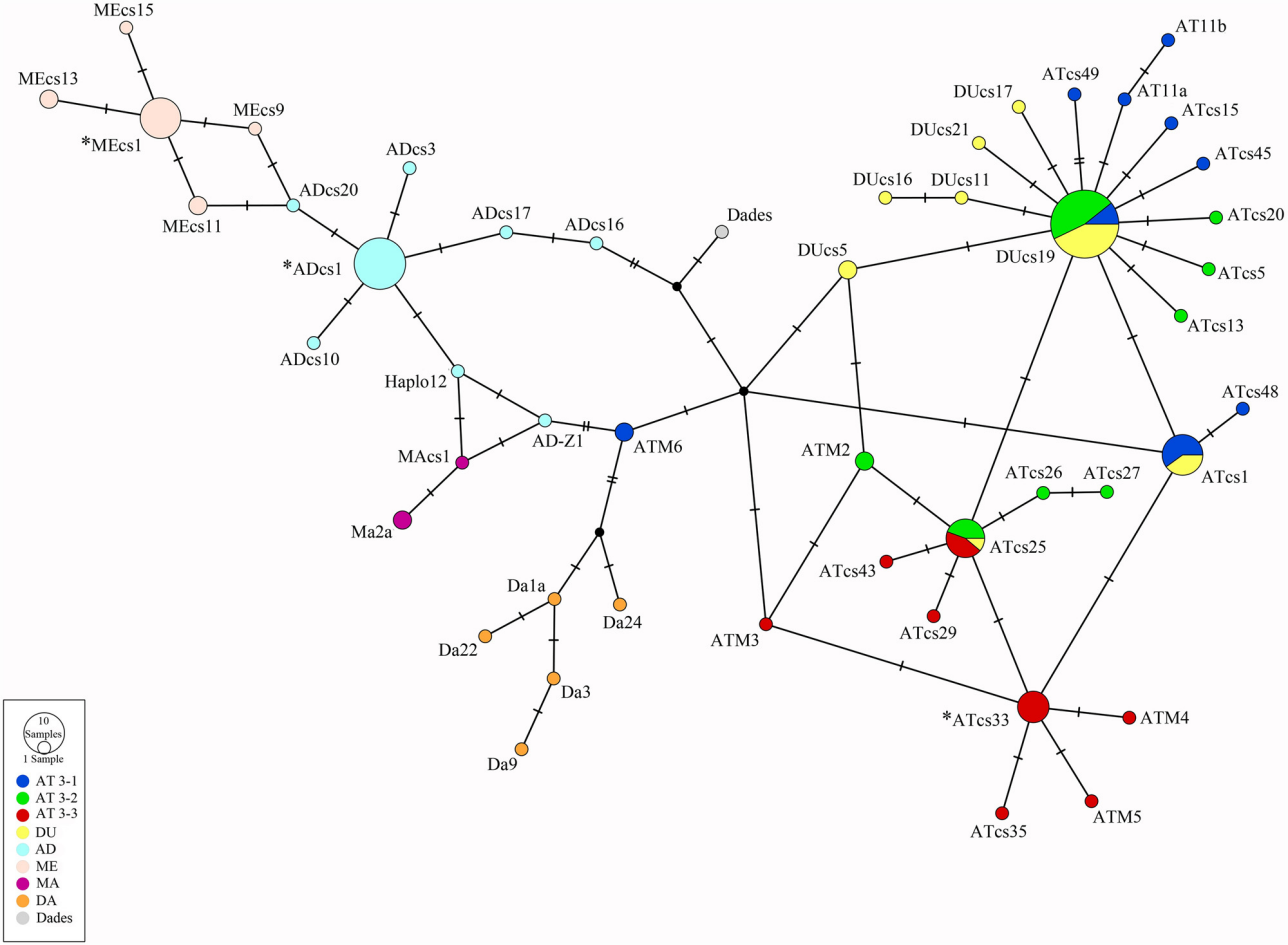
Statistical parsimony haplotypes network. Haplotype network showing the relationships between modern haplotypes and haplotypes observed in the archaeological samples analysed in the present study (indicated by asterisks).

The above classification was also confirmed by a statistical parsimony haplotype network (Fig 4). In fact, the three haplotypes detected from ancient trout were classified into three different clusters. The nucleotide sequences obtained by the specimens V62, M62 and M57 were identical to the partial haplotype MEcs-1. In addition, the haplotype MEcs-1 occupied a central position in a star like structure characterizing the ME lineage. In a similar manner, the specimens PT57 showed a nucleotide sequence identical to the partial haplotype ADcs-1. Also in this case, we detected this haplotype in the centre of a star like figure for the AD lineage. Finally, the specimens Pr50, M50, M48 and V46, showed the same nucleotide composition of the partial haplotype ATcs-33. In the parsimony network this latter haplotype represented the basal haplotype of a star-like cluster composed by the haplotypes already classified by Cortey et al. [19] into the southern Atlantic clade AT3-3.

### Correlation of DNA data to Greenland Ice-Core (GRIP)

**Fig 5:**
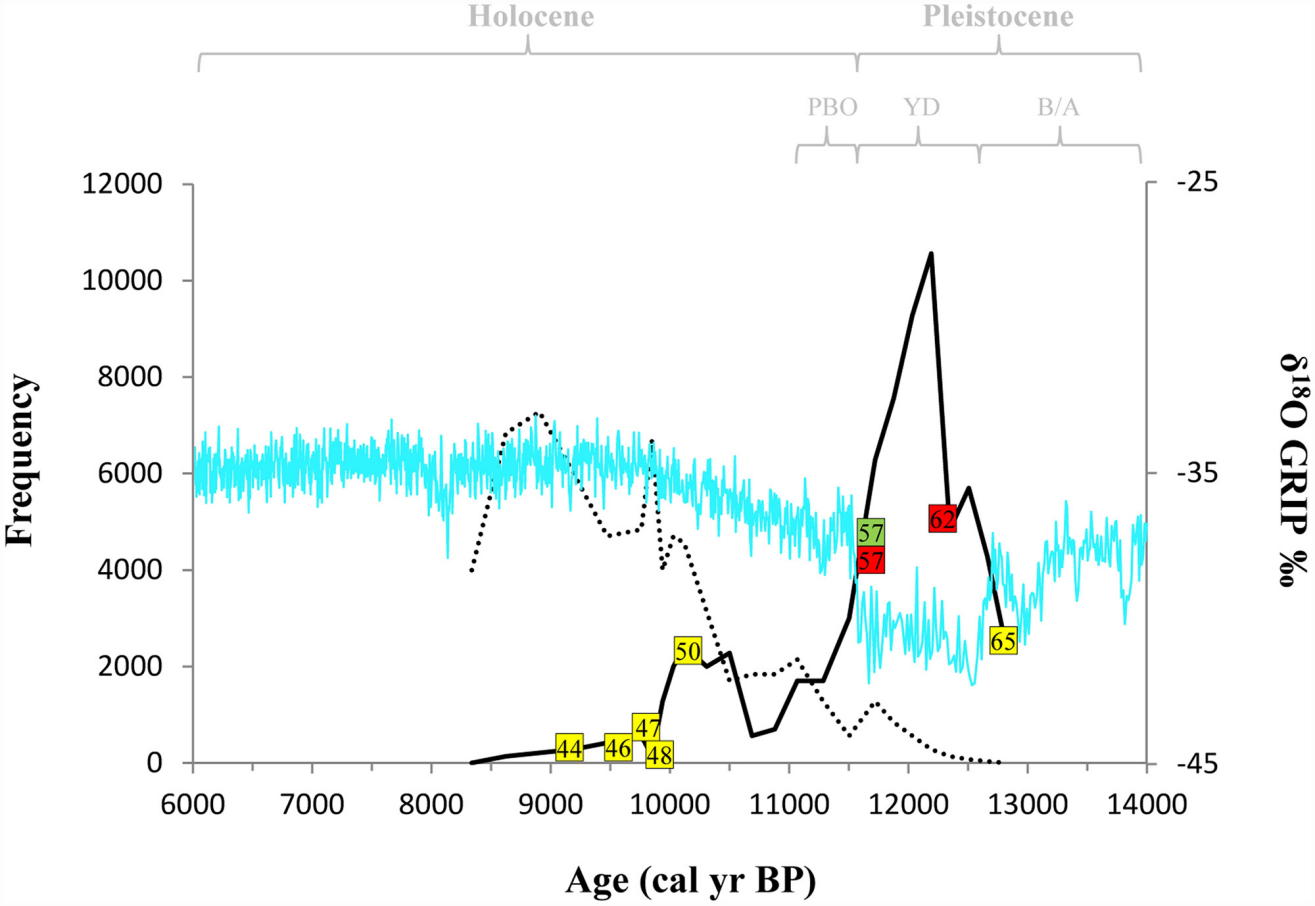
Relationship between abundance of remains, mitochondrial haplotypes observed in this study and Pleistocene-Holocene climatic fluctuations. The continuous line in the graph indicates the frequency of *Salmo trutta* remains and the dotted line indicates the frequency of *Patella* and *Monodonta* remains in all sections of the stratigraphic succession of GSM (approximate reconstruction from [40]). Mitochondrial haplotypes observed in this study are indicated by coloured squares: ATcs-33 in yellow, ADcs-1 in green and MEcs-1 in red. This information is related with the δ^18^O (‰) climate proxies of the GRIP ice core record represented by a blue line (dataset from NOAA website: http://www1.ncdc.noaa.gov/pub/data/paleo/icecore/greenland/summit/grip/isotopes/gripd18o.txt).

The CalPal calibration (Table 4) allowed to refine the age of the interval studied (10,850 and 7,555 years BP, not calibrated) between 12,820 and 8,340 cal yr BP. The correlation between our DNA data and the paleoclimatic reconstruction based on the δ18O of the Greenland Ice-Core (GRIP) is shown in Fig 5. The comparison with the paleo-temperature shows that our samples encompass three different climatic phases: i) the Bølling/Allerød (warm and wet); ii) the Younger Dryas (cold and dry, hereafter YD); and iii) the Preboreal oscillation (a climate reversal) (e.g., [55]), which mark the transition from Pleistocene to Holocene. In particular, in the oldest sample (TP65, about 12,820 cal yr BP), which falls within an interval characterized by a transition from warm temperature (as evidenced by low δ^18^O values, related to the Bølling/Allerød interstadial) to the start of the cold YD, we observed the haplotype ATcs-33 (Table 4 and Fig 5). In more recent strata, we observed the haplotypes MEcs-1 (samples: V62, M62 and M57) and ADcs-1 (PT57) (Table 4 and Fig 5). The time interval of these strata correlated to the YD cold stadial (about 12,800–11,600 cal yr BP) and probably to the Preboreal Oscillation, a climate reversal due to the melt water flow originated after the abrupt drainage of glacial Lake Agassiz [55] which occurred about 11,335 cal yr BP, and lasted until 10,750 cal yr BP. Finally, from about 10,120 cal yr BP to the disappearance of trout remains (layers IX and VIII, sections 45–46 and 40–41, about 9,760 and 8,340 cal yr BP) we detected only the haplotype ATcs-33 (samples: Pr50, M50, M48, M47, V46 and V44) (Table 4 and Fig 5). This last interval identifies the beginning of the warmer Holocene climate persisting nowadays.

**Table 4:** Radiocarbon dates and CalPal calibration results.

Layer	Section	Sample type	Sample name	AGE yr BP	AGE cal yr BP	95% range cal BP
VIII	40-41	Charcoal	R-285	7,555 ± 85	**8,340**	8,520–8,160
IXa	45-46	Charred bones	R-188	9,070 ± 80	**10,250**	10,450–10,050
IXb	45-46	Charcoal	R-187	8,735 ± 80	**9,760**	10,040–9,480
IXb	45-46	Insoluble residue of R-187	R-187α	8,875 ± 85	**9,960**	10,280–9,640
X	49-50a	Charcoal	R-286	9,020 ± 125	**10,120**	10,500–9,740
X	49-50b	Burnt bones	R-287	9,035 ± 100	**10,140**	10,460–9,820
X	49-50c	*Porchus turbinatus*	R-288	8,600 ± 120	**9,180**	9,480–8,880
X	49-50d	*Patella* shells	R-288A	9,800 ± 140	**10,700**	11,100–11,300
X	54-55a	Charcoal	R-289	10,300 ± 100	**12,140**	12,640–11,640
X	54-55b	Burnt bones	R-290	9,750 ± 100	**11,070**	11,410–10,730
X	54-55c	Shells marine molluscs(mainly *Patella* shells)	R-291	10,450 ± 100	**11,530**	11,950–11,110
Xa	57-58	Charred bones	R-186	10,030 ± 90	**11,570**	11,950–11,190
Xb	57-58	Charcoal	R-185	10,120 ± 70	**11,720**	12,120–11,320
X	64-65	Charcoal	R-292	10,850 ± 100	**12,820**	13,000–12,640
X	71-72	Charcoal	R-293	12,100 ± 150	**14,200**	14,820–13,580

Details and radiocarbon dates of samples from different layers and sections of the stratigraphic succession from GSM e.g., [42, 43]. In bold, the results of radiocarbon dates calibration using CalPal software [44], CalCurve: CalPal 2007 HULU or INTCAL 04 Marine [45].

## Discussion

### Validation of the results and criticisms

The main criticisms to this study might relate to: i) the small sample size and ii) the scarcity and degradation of extracted DNA from archaeological samples. As for the first issue, obtaining samples from which aDNA can be extracted is challenging, especially because during laboratory analysis the samples must be destroyed. Consequently, the limited availability of larger sample sizes is unavoidable in such studies. As for the second issue, we retain our results reliable as: i) we avoided contamination during DNA extraction and PCR by mean of a specific decontamination protocol (see material and methods), ii) we obtained comparable haplotypes from different samples of the same layer and from different extraction replicas of the same samples, iii) we observed the haplotype ATcs-33 never observed before in the modern samples analysed in our laboratories (in case of contamination we would expect to find the haplotype ATcs-1, or similar domestic haplotypes) and iv) the absence of new mutations and/or chimera sequences suggests that the sequence analysis presented in this study is not altered by the typical PCR errors generated by aDNA lesions.

### mtDNA diversity of the Paleolithic Mediterranean trout

The analysis of genetic diversity of mtDNA extracted from skeletal remains of trout revealed the presence of three different brown trout phylogenetic lineages: ME, AD and AT. In particular, for the lineages ME and AD we observed the basal haplotypes (MEcs-1 and ADcs-1) of both lineages in agreement with the wide geographical distribution of these mtDNA variants shown by previous studies [17, 54, 56]. According to Cortey et al. [54], the wide geographic distribution of MEcs-1 and ADcs-1 and their location at central position in star-like phylogenies fit well with a sudden demographic expansion for both lineages. As an example, paleontological data from the Mediterranean area show that salmonids varied their range by activating migratory tactics in colder climatic periods. In particular, the study of fossil remains from Iberian Peninsula suggested that during glacial intervals brown trout occupied river portions in lowland areas currently dominated by thermophilic fish species [34]. This example suggests a similar scenario during the Pleistocene to explain the natural presence of the AT lineage in southern Italy. In fact, the haplotype ATcs-33, belonging to the so called "southern Atlantic clade" AT3-3, [18] and already observed in Mediterranean rivers (Spain, North Africa and Sicily, [19, 56, 57]), was detected in the present study. This haplotype also shows a central position in a star-like phylogenetic network and a pattern of nucleotide diversity indicating a relatively recent origin, about 100,000 years ago [56]. We therefore suggest that the beginning of the last glacial interval represented an important opportunity for this pioneer clade to expand its range in the Mediterranean basin. The recovery of this Atlantic haplotype among Upper Paleolithic remains of GSM shows that the expansion of the southern clade AT3-3 was not limited to the rivers of South-eastern Sicily; instead it moved northwards in the Mediterranean basin.

The results of this study also show that different haplotypes occur in different sections and layers of the stratigraphic succession. The oldest samples here analysed (about 12,820 cal yr BP) were characterized by the presence of haplotype ATcs-33. The haplotype composition changed between about 12,800–11,600 cal yr BP when only the "Mediterranean" haplotypes MEcs-1 and ADcs-1 were found. Finally, in the most recent layers (about 10,120–8,340 cal yr BP), we detected only haplotype ATcs-33 (Fig 5). The changes in the haplotype composition observed (from ATcs-33 to MEcs-1 and ADcs-1, and *vice versa*) along the stratigraphic succession coincide with the change in the abundance pattern of *S*. *trutta* remains described by Durante [40]. In fact, the most abundant deposit of bone remains coincide with the observation of ME and AD haplotypes that in turn is related to the sudden onset of the YD (Fig 5). However, the limited number of specimens analysed in this study do not allow us to outline strong and conclusive hypothesis concerning the paleo-ecologic dynamics occurring on *S*. *trutta* populations from southern Italian peninsula. Therefore, future analyses of a larger number of samples will allow to make sound inferences on the paleo-ecologic dynamic occurring on *S*. *trutta* populations from southern Italian Peninsula.

## Concluding remarks

This is the first aDNA study performed on brown trout sub-fossil bone remains. Here, we analysed remains dating back to the Pleistocene-Holocene transition [39], and we observed a probably correspondence between the frequency of *S*. *trutta* in the deposit and variation in mitochondrial genetic diversity. Although the scarce number of bones analysed call for caution on results interpretation we tentatively relate this variation in the genetic pattern to changes in the ecological and demographic characteristics resulting after the abrupt and short climatic event known as the "Younger Dryas" (Pleistocene/Holocene transition). The results discussed highlight how the aDNA from sub-fossil remains of salmonids can provide crucial information to link population processes with the climatic changes.

If the results of this study suggest that trout survived in the Mediterranean basin during the alternate climatic phases of Pleistocene, then we have to seriously consider the current deterioration in environmental conditions mediated by humans. Nowadays, the survival chances for modern and/or future populations to severe warming periods could be even more challenging than before because of anthropogenic factors, such as the introduction of alien taxa, freshwater habitat alteration and excessive water abstraction (e.g., [58–60]). This study points out the importance of the archaeological DNA data to understand the complex dynamics of colonization that characterized many Holarctic species in response to climate changes which occurred during the Pleistocene. The need for an improvement of our knowledge on how species respond to climatic changes is particularly important for freshwater fishes and in particular for salmonids, especially if we take into account the current global warming and the enormous socioeconomics interests surrounding the management of local trout populations (e.g., [61]).

## Supporting Information

S1 Table Details regarding the mtDNA D-loop sequences using to obtain the statistical parsimony network. (DOCX)

S2 Table Amplification success. (DOCX)
