# Supplementary material for: The Effects of Paleoclimatic Events on Mediterranean Trout: Preliminary Evidences from Ancient DNA
Source: PLoS One. 2016 Jun 22;11(6):e0157975. doi: 10.1371/journal.pone.0157975 (PMC4917132; doi:10.1371/journal.pone.0157975)
Supplement: S1 Table — (DOCX) [file pone.0157975.s001.docx]

**S1 Table. Details regarding the mtDNA D-loop sequences using to obtain the statistical parsimony network.**

| **Haplotype** | **GenBank Accession no.** | **Reference** |
| --- | --- | --- |
| ADcs1 | AY836330 | Cortey et al., 2004 |
| ADcs2 | [AY836331](http://www.ncbi.nlm.nih.gov/nuccore/AY836331.1) | Cortey et al., 2004 |
| ADcs3 | [AY836332](http://www.ncbi.nlm.nih.gov/nuccore/AY836332.1) | Cortey et al., 2004 |
| ADcs4 | [AY836333](http://www.ncbi.nlm.nih.gov/nuccore/AY836333.1) | Cortey et al., 2004 |
| ADcs5 | [AY836334](http://www.ncbi.nlm.nih.gov/nuccore/AY836334.1) | Cortey et al., 2004 |
| ADcs6 | [AY836335](http://www.ncbi.nlm.nih.gov/nuccore/AY836335.1) | Cortey et al., 2004 |
| ADcs7 | [AY836336](http://www.ncbi.nlm.nih.gov/nuccore/AY836336.1) | Cortey et al., 2004 |
| ADcs8 | [AY836337](http://www.ncbi.nlm.nih.gov/nuccore/AY836337.1) | Cortey et al., 2004 |
| ADcs9 | [AY836338](http://www.ncbi.nlm.nih.gov/nuccore/AY836338.1) | Cortey et al., 2004 |
| ADcs10 | [AY836339](http://www.ncbi.nlm.nih.gov/nuccore/AY836339.1) | Cortey et al., 2004 |
| ADcs11 | [AY836340](http://www.ncbi.nlm.nih.gov/nuccore/AY836340.1) | Cortey et al., 2004 |
| ADcs12 | [AY836341](http://www.ncbi.nlm.nih.gov/nuccore/AY836341.1) | Cortey et al., 2004 |
| ADcs13 | [AY836342](http://www.ncbi.nlm.nih.gov/nuccore/AY836342.1) | Cortey et al., 2004 |
| ADcs14 | [AY836343](http://www.ncbi.nlm.nih.gov/nuccore/AY836343.1) | Cortey et al., 2004 |
| ADcs15 | [AY836344](http://www.ncbi.nlm.nih.gov/nuccore/AY836344.1) | Cortey et al., 2004 |
| ADcs16 | [AY836345](http://www.ncbi.nlm.nih.gov/nuccore/AY836345.1) | Cortey et al., 2004 |
| ADcs17 | [AY836346](http://www.ncbi.nlm.nih.gov/nuccore/AY836346.1) | Cortey et al., 2004 |
| ADcs18 | [AY836347](http://www.ncbi.nlm.nih.gov/nuccore/AY836347.1) | Cortey et al., 2004 |
| ADcs19 | [AY836348](http://www.ncbi.nlm.nih.gov/nuccore/AY836348.1) | Cortey et al., 2004 |
| ADcs20 | [AY836349](http://www.ncbi.nlm.nih.gov/nuccore/AY836349.1) | Cortey et al., 2004 |
| AD-Z1 | DQ381565 | Susnik et al., 2007 |
| Haplo12 | AY926570 | Susnik et al., 2006 |
| Ma2a | DQ841189 | Meraner et al., 2007 |
| Ma2b | DQ841190 | Meraner et al., 2007 |
| MAcs1 | AY836365 | Cortey et al., 2004 |
| MEcs1 | [AY836350](http://www.ncbi.nlm.nih.gov/nuccore/AY836350.1) | Cortey et al., 2004 |
| MEcs2 | [AY836351](http://www.ncbi.nlm.nih.gov/nuccore/AY836350.1) | Cortey et al., 2004 |
| MEcs3 | [AY836352](http://www.ncbi.nlm.nih.gov/nuccore/AY836350.1) | Cortey et al., 2004 |
| MEcs4 | [AY836353](http://www.ncbi.nlm.nih.gov/nuccore/AY836350.1) | Cortey et al., 2004 |
| MEcs5 | [AY836354](http://www.ncbi.nlm.nih.gov/nuccore/AY836350.1) | Cortey et al., 2004 |
| MEcs6 | [AY836355](http://www.ncbi.nlm.nih.gov/nuccore/AY836350.1) | Cortey et al., 2004 |
| MEcs7 | [AY836356](http://www.ncbi.nlm.nih.gov/nuccore/AY836350.1) | Cortey et al., 2004 |
| MEcs8 | [AY836357](http://www.ncbi.nlm.nih.gov/nuccore/AY836350.1) | Cortey et al., 2004 |
| MEcs9 | [AY836358](http://www.ncbi.nlm.nih.gov/nuccore/AY836350.1) | Cortey et al., 2004 |
| MEcs10 | [AY836359](http://www.ncbi.nlm.nih.gov/nuccore/AY836350.1) | Cortey et al., 2004 |
| MEcs11 | [AY836360](http://www.ncbi.nlm.nih.gov/nuccore/AY836350.1) | Cortey et al., 2004 |
| MEcs12 | [AY836361](http://www.ncbi.nlm.nih.gov/nuccore/AY836350.1) | Cortey et al., 2004 |
| MEcs13 | [AY836362](http://www.ncbi.nlm.nih.gov/nuccore/AY836350.1) | Cortey et al., 2004 |
| MEcs14 | [AY836363](http://www.ncbi.nlm.nih.gov/nuccore/AY836350.1) | Cortey et al., 2004 |
| MEcs15 | [AY836364](http://www.ncbi.nlm.nih.gov/nuccore/AY836350.1) | Cortey et al., 2004 |
| ATcs1 | AF273086 | Cortey and Garcia-Marin, 2000 |
| ATcs2 | AF273087 | Cortey and Garcia-Marin, 2000 |
| ATcs3 | AF274574 | Cortey and Garcia-Marin, 2000 |
| ATcs4 | AF274575 | Cortey and Garcia-Marin, 2000 |
| ATcs5 | AF274576 | Cortey and Garcia-Marin, 2000 |
| ATcs6 | AF274577 | Cortey and Garcia-Marin, 2000 |
| ATcs11 | [AY836327](http://www.ncbi.nlm.nih.gov/nuccore/AY836327.1) | Cortey et al., 2004 |
| ATcs12 | [AY836328](http://www.ncbi.nlm.nih.gov/nuccore/AY836327.1) | Cortey et al., 2004 |
| ATcs13 | [AY836329](http://www.ncbi.nlm.nih.gov/nuccore/AY836327.1) | Cortey et al., 2004 |
| ATcs14 | EF530476 | Cortey et al., 2009 |
| ATcs15 | EF530477 | Cortey et al., 2009 |
| ATcs16 | EF530478 | Cortey et al., 2009 |
| ATcs17 | EF530479 | Cortey et al., 2009 |
| ATcs18 | EF530480 | Cortey et al., 2009 |
| ATcs19 | EF530481 | Cortey et al., 2009 |
| ATcs20 | EF530482 | Cortey et al., 2009 |
| ATcs21 | EF530483 | Cortey et al., 2009 |
| ATcs22 | EF530484 | Cortey et al., 2009 |
| ATcs23 | EF530485 | Cortey et al., 2009 |
| ATcs24 | EF530486 | Cortey et al., 2009 |
| ATcs25 | EF530487 | Cortey et al., 2009 |
| ATcs26 | EF530488 | Cortey et al., 2009 |
| ATcs27 | EF530489 | Cortey et al., 2009 |
| ATcs28 | EF530490 | Cortey et al., 2009 |
| ATcs29 | EF530491 | Cortey et al., 2009 |
| ATcs30 | EF530492 | Cortey et al., 2009 |
| ATcs31 | EF530493 | Cortey et al., 2009 |
| ATcs32 | EF530494 | Cortey et al., 2009 |
| ATcs33 | EF530495 | Cortey et al., 2009 |
| ATcs34 | EF530496 | Cortey et al., 2009 |
| ATcs35 | EF530497 | Cortey et al., 2009 |
| ATcs36 | EF530498 | Cortey et al., 2009 |
| ATcs37 | EF530499 | Cortey et al., 2009 |
| ATcs38 | EF530500 | Cortey et al., 2009 |
| ATcs39 | EF530501 | Cortey et al., 2009 |
| ATcs41 | EF530502 | Cortey et al., 2009 |
| ATcs42 | EF530503 | Cortey et al., 2009 |
| ATcs43 | EF530504 | Cortey et al., 2009 |
| ATcs45 | EF530505 | Cortey et al., 2009 |
| ATcs46 | EF530506 | Cortey et al., 2009 |
| ATcs47 | EF530507 | Cortey et al., 2009 |
| ATcs48 | EF530508 | Cortey et al., 2009 |
| ATcs49 | EF530509 | Cortey et al., 2009 |
| ATcs50 | EF530510 | Cortey et al., 2009 |
| ATcs51 | EF530511 | Cortey et al., 2009 |
| ATcs52 | EF530512 | Cortey et al., 2009 |
| AT11a | AY185578 | Duftner et al., 2003 |
| AT11b | AY185579 | Duftner et al., 2003 |
| At1e | DQ841192 | Meraner et al., 2007 |
| ATM1 | JF297978 | Snoj et al., 2011 |
| ATM2 | JF297979 | Snoj et al., 2011 |
| ATM3 | JF297980 | Snoj et al., 2011 |
| ATM4 | JF297975 | Snoj et al., 2011 |
| ATM5 | JF297977 | Snoj et al., 2011 |
| ATM6 | JF297976 | Snoj et al., 2011 |
| ATM7 | JF297982 | Snoj et al., 2011 |
| Dades | JF297981 | Snoj et al., 2011 |
| Da1a | AY185568 | Duftner et al., 2003 |
| Da3 | AY185571 | Duftner et al., 2003 |
| Da9 | AY185572 | Duftner et al., 2003 |
| Da22 | AY185573 | Duftner et al., 2003 |
| Da24 | AY185576 | Duftner et al., 2003 |
| DUcs1 | EF530513 | Snoj et al., 2011 |
| DUcs2 | EF530514 | Snoj et al., 2011 |
| DUcs3 | EF530515 | Snoj et al., 2011 |
| DUcs4 | EF530516 | Snoj et al., 2011 |
| DUcs5 | EF530517 | Snoj et al., 2011 |
| DUcs6 | EF530518 | Snoj et al., 2011 |
| DUcs7 | EF530519 | Snoj et al., 2011 |
| DUcs8 | EF530520 | Snoj et al., 2011 |
| DUcs9 | EF530521 | Snoj et al., 2011 |
| DUcs10 | EF530522 | Snoj et al., 2011 |
| DUcs11 | EF530523 | Snoj et al., 2011 |
| DUcs12 | EF530524 | Snoj et al., 2011 |
| DUcs13 | EF530525 | Snoj et al., 2011 |
| DUcs14 | EF530526 | Snoj et al., 2011 |
| DUcs15 | EF530527 | Snoj et al., 2011 |
| DUcs16 | EF530528 | Snoj et al., 2011 |
| DUcs17 | EF530529 | Snoj et al., 2011 |
| DUcs18 | EF530530 | Snoj et al., 2011 |
| DUcs19 | EF530531 | Snoj et al., 2011 |
| DUcs20 | EF530532 | Snoj et al., 2011 |
| DUcs21 | EF530533 | Snoj et al., 2011 |
| DUcs22 | EF530534 | Snoj et al., 2011 |
| DUcs23 | EF530535 | Snoj et al., 2011 |
