# Supplementary material for: The Effects of Paleoclimatic Events on Mediterranean Trout: Preliminary Evidences from Ancient DNA
Source: PLoS One. 2016 Jun 22;11(6):e0157975. doi: 10.1371/journal.pone.0157975 (PMC4917132; doi:10.1371/journal.pone.0157975)
Supplement: S2 Table — (DOCX) [file pone.0157975.s002.docx]

S2 Table. Amplification success.

| **Primer pairs** | | | | | | | | |
| --- | --- | --- | --- | --- | --- | --- | --- | --- |
| **Sample ID** | **StCR-1** | **StCR-2a** | **StCR-2c*** | **StCR-2b** | **StCR-3** | **StCR-4** | **StCR-5** | **StCR-7** |
| **First set of samples analysed** | | | | | | | | |
| **V44** | NA | NA | NA | . | NA | . | . | NA |
| **V46** | . | . |  | . | . | . | . | . |
| **M50** | . | NA | . | . | . | . | . | . |
| **PT57** | . | NA | NA | . | . | NA | . | . |
| **M62** | . | . |  | . | . | . | . | . |
| **TP65** | . | NA | . | . | NA | NA | . | . |
| **Second set of samples analysed** | | | | | | | | |
| **M47** | NA | NA | . | . | . | . | . | . |
| **M48** | . | . |  | . | . | . | . | . |
| **Pr50** | . | . |  | . | . | . | . | . |
| **M57** | . | . |  | . | . | . | . | . |
| **V62** | . | . |  | . | . | . | . | . |
| **Pr65** | NA | NA | NA | NA | NA | NA | NA | NA |

Sequences obtained for each primer pair used. The dots indicate all fragments successfully amplified and sequenced. The acronym “NA” indicates not amplified.

*The StCR-2c primer pair was used only in case of non-amplification with StCR-2a.
